# Avoiding Packing When an External Fixator Is Used in Cervical Cancer Brachytherapy: The Rationale for Vaginal Gauze Packing on Rectum and Bladder

**DOI:** 10.7759/cureus.71936

**Published:** 2024-10-20

**Authors:** Ibrahim Halil Suyusal, Aykut Oğuz Konuk, Umut Diremsizoglu, Onur Ari, Hasan Furkan Cevik, Nezihan Topal, Aysegül Ucuncu Kefeli, Emine Binnaz Sarper, Maksut Gorkem Aksu

**Affiliations:** 1 Radiation Oncology, Kocaeli University Faculty of Medicine, Kocaeli, TUR

**Keywords:** bladder protection, external fixation, rectum protection, uterine cervical cancer, vaginal gauze packing

## Abstract

Purpose: The aim of this study is to investigate the rational effects of packing, which has seen reduced use after the introduction of fixators that perform the stabilization function-on rectum, bladder, and A point doses when used in conjunction with fixators.

Methods: A retrospective analysis was conducted on inoperable-cervical-cancer patients who underwent brachytherapy with tandem and ovoid at Kocaeli University Hospital between January 2023 and May 2024. Patients received external beam radiotherapy (EBRT), followed by high-dose-rate brachytherapy with either standard or packed plans. In all patient plans, a perineal bar served as the external fixator. Dosimetric outcomes for the rectum and bladder were compared, focusing on D0.1 cc, D1 cc, D2 cc, D5 cc, and D10 cc dose parameters.

Results: A total of 34 patients and 68 plans were compared. Each patient’s standard plan (SP) and vaginal gauze packing (VGP) plan were compared. In both plans, the doses to the right and left A points were kept identical. VGP plan significantly reduced rectal doses by 23.79% for D0.1 cc, 22.94% for D1 cc, 22.20% for D2 cc, 20.29% for D5 cc, and 18.43% for D10 cc. No significant differences were observed in bladder dose parameters between the two plans. The rectal volumes were similar in both groups.

Conclusion: VGP effectively reduces rectal radiation exposure during cervical cancer brachytherapy, providing a cost-effective and feasible protective measure. Even when an external fixator is used, packing should not be discontinued. However, additional strategies may be needed to optimize bladder protection. Further research with larger patient cohorts and long-term follow-ups are recommended to enhance clinical practice.

## Introduction

Cervical cancer is one of the most common types of cancer among women worldwide and poses a significant health problem, particularly in developing countries [1]. In cases of cervical cancer detected at an early stage, treatment methods such as surgery and radiotherapy are highly effective [2]. Intracavitary brachytherapy delivers high doses of radiation directly to the tumor while minimizing exposure to surrounding healthy tissues, making it particularly useful for inoperable tumors [3]. However, during brachytherapy, healthy organs such as the bladder and rectum may also be exposed to radiation, increasing the risk of side effects [4]. Therefore, protecting the bladder and rectum from radiation is crucial.

Vaginal gauze packing (VGP) is generally used to stabilize the applicator and based on the idea that it protects the rectum and bladder with its anatomical barrier effect. Since the applicator cannot be stabilized without the use of an external fixator, packing becomes mandatory. However, since the introduction of external fixators, there has been an opportunity to receive treatment without packing. In this case, it is essential to better understand and investigate the effects of VGP on the bladder and rectum. After the development of fixators that perform the stabilization function, there is a question of how much packing reduces doses to the rectum and bladder when not used for fixation, and whether this reduction has statistical significance. In the literature, the lack of data regarding this issue is increasingly questioned. Within this context, a rational basis is sought for the continuation of the application. The aim of this study is to investigate the rational effects of packing on rectum and bladder doses in two groups: one that utilizes VGP with fixators and one that does not use VGP after the introduction of fixators that perform the stabilization function.

A study conducted in 2016 suggests that vaginal packing during brachytherapy may be a potential solution for reducing doses to the rectum and bladder [5]. Among the methods used, gauze packing is easily accessible and cost effective, reducing the doses received by organs at risk and also decreasing the risk of uterine perforation [6,7]. In the recommendations published in 2023 by the Spanish Brachytherapy Group of the Spanish Society of Radiation Oncology and the Spanish Society of Medical Physics, the use of an external fixator attached to the patient is recommended to prevent the applicator from moving [8].

The study aims to make significant contributions to clinical practice by enhancing the safety of brachytherapy applications and optimizing treatment success with the help of newly developed auxiliary devices. The findings of this research will facilitate the development of more effective and safer methods for the treatment of cervical cancer.

## Materials and methods

Patient selection

All inoperable cervical cancer patients treated with brachytherapy at Kocaeli University Hospital between January 2023 and May 2024 participated in the study. The selected patients had at least two planning CT scans, one with vaginal packing and one without. In both plans for all patients, a perineal bar served as the external fixator. All patients were diagnosed with inoperable cervical cancer at FIGO stage 2B-3C. The Ethics Committee of the university approved the study protocol (project number: 2024/125).

All patients received 50.4 cGy in 28 fractions of external beam radiotherapy (EBRT), along with four-six cycles of concurrent weekly cisplatin. Post EBRT, patients underwent contrast-enhanced MRI for evaluation before brachytherapy. All patients received high-dose-rate (HDR) brachytherapy under general anesthesia with a Fletcher-Suit-Delclos tandem ovoid applicator. Plans were created to deliver 600 cGy per fraction to point A over four fractions, administered twice weekly.

Applicator placement and vaginal packing

Patients were taken into the procedure under general anesthesia with pain control ensured. Initially, a gynecological examination was performed to assess the anatomical structure and local tumor spread.

Standard planning involved placing patients in the lithotomy position with a Foley catheter, administering 55 cc saline and 5 cc contrast medium to the bladder and inflating the balloon with 7 cc contrast. The rectum was administered with 25 cc saline and 5 cc contrast medium. Tandem length was determined based on uterine size using imaging and examination, and appropriate tandem lengths of 4, 6, or 8 cm were selected. The widest possible ovoid diameter was chosen from available sizes (2, 2.5, and 3 cm). Finally, the placed applicators were secured to the patient using a perineal bar. CT scans were obtained for three-dimensional (3D) imaging.

**Figure 1:**
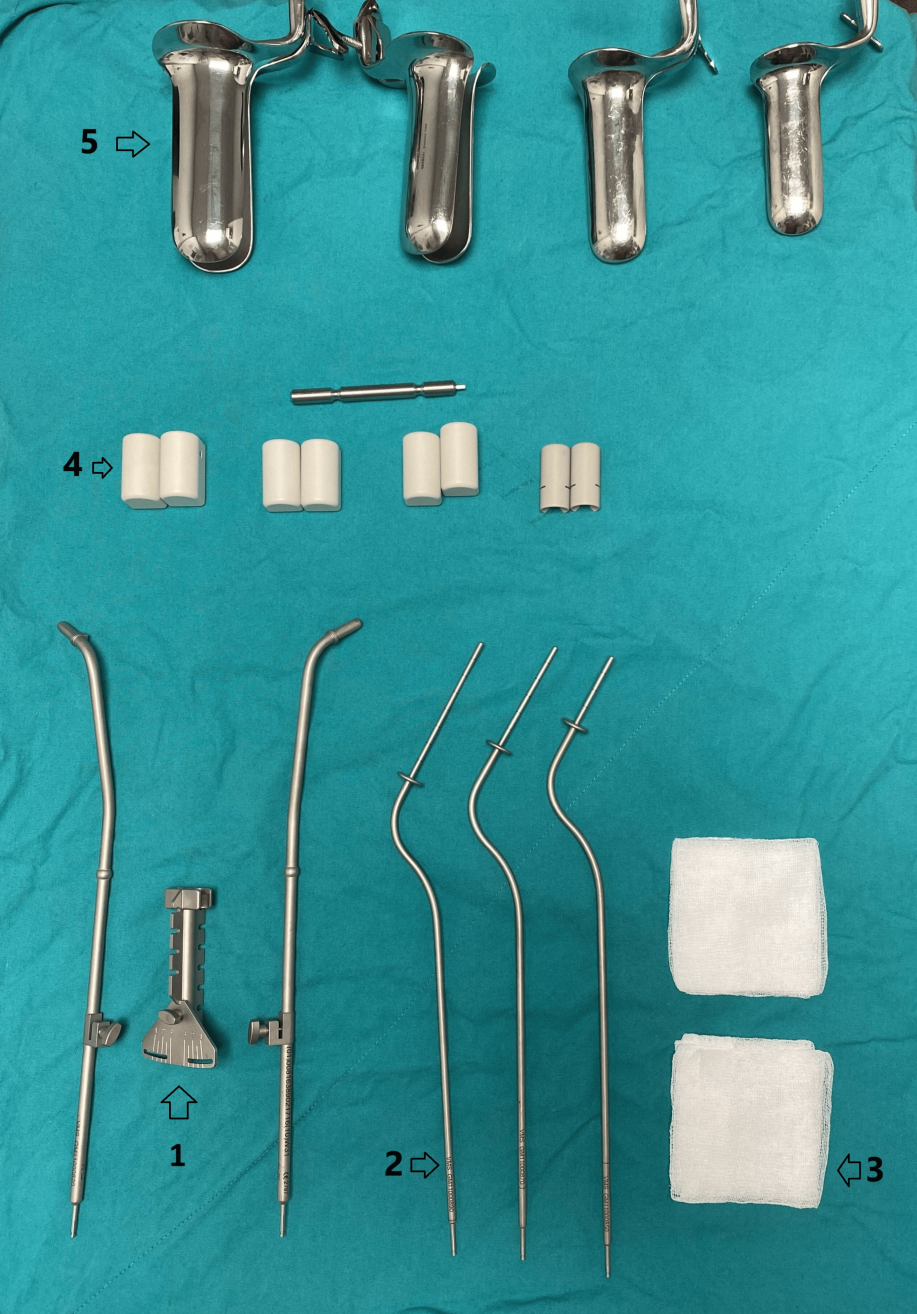
Materials used in the study 1. Tandem stabilizer with ovoid stabilizers on both sides; 2. Tandems measuring 8 cm, 6 cm, and 4 cm in length from left to right; 3. Two pieces of gauze; 4. Ovoids with diameters of 30 mm, 25 mm, 20 mm, and 15 mm from left to right; 5. From left to right: a large-sized speculum, a medium-sized speculum, a small-sized speculum, and on the far right a short, small speculum for patients with a shallow vagina.

For the VGP plan, gauze soaked in diluted radio-opaque solution (1/10 saline) was placed posterior and anterior to the tandem and ovoids, extending to the introitus. The number of gauzes varied depending on vaginal size and flexibility, with the goal of maximizing distance from the rectum and bladder (Figure 1). In this plan as well, the placed applicators were secured to the patient using a perineal bar, and CT scans were obtained.

Brachytherapy planning and dose comparison

Treatment planning was applied uniformly to both CT images, with doses planned to deliver 600 cGy to point A [9]. Doses to the D2 cc of the bladder and rectum were optimized to not exceed 80% and 70% of the prescribed dose, respectively.

**Figure 2:**
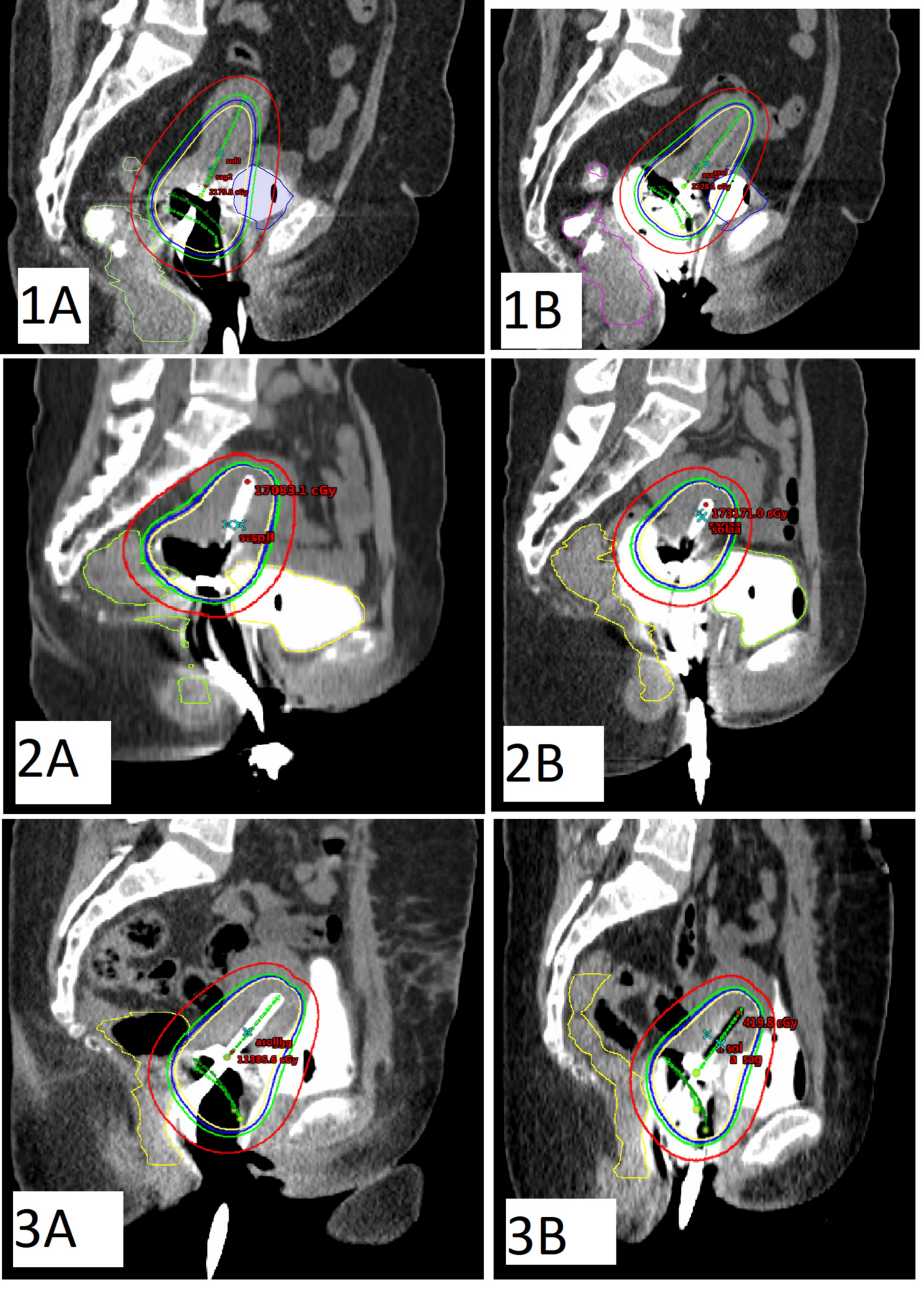
Sagittal CT images of the SP and VGP plan for three patients The images labeled with “A” next to the patient numbers correspond to the SP, while those labeled with “B” correspond to the VGP plan. Dose distributions: the yellow line represents 100% isodose; the blue line represents 90% isodose; the green line represents 80% isodose; and the red line represents 50% isodose. SP: Standard plan; VGP: Vaginal gauze packing

Bladder volumes were contoured consistently across all patients. Rectal volumes were measured to assess volumetric differences between scans. Dosimetric comparisons of D0.1 cc, D1 cc, D2 cc, D5 cc, and D10 cc were made for both the bladder and rectum. The sagittal CT images and isodose curves of the SP and VGP plan for three patients are shown in Figure 2.

**Figure 3:**
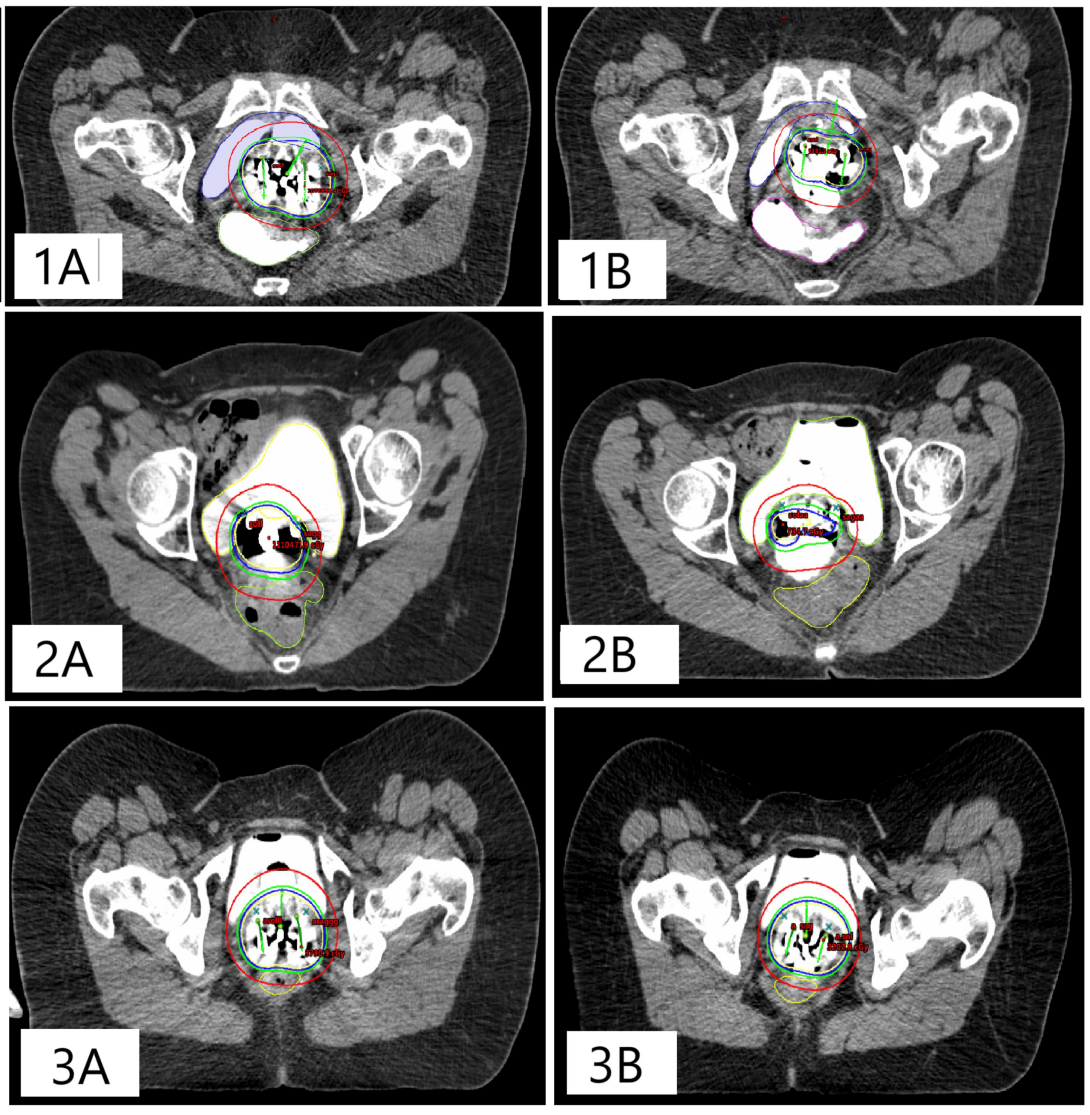
Axial CT images of SP and VGP plan for the three patients The images labeled with “A” next to the patient numbers correspond to the SP, while those labeled with “B” correspond to the VGP plan. Dose distributions: the yellow line represents 100% isodose; the blue line represents 90% isodose; the green line represents 80% isodose; and the red line represents 50% isodose. SP: Standard plan; VGP: Vaginal gauze packing

The axial sections and isodose curves of the same patients are also shown in Figure 3.

Data were expressed as mean ± standard deviation and analyzed using paired t-tests with SPSS 25.0. A p-value of <0.05 was considered statistically significant.

## Results

Patient characteristics are summarized in Table 1. 34 patients who met the inclusion criteria were included. The median age was 53 years. All patients received 600 cGy to point A using tandem and ovoid. 16 patients were FIGO Stage 2B, six patients were Stage 3B, and 12 patients were Stage 3C. Packing was performed using two-five pieces of gauze, with an average of three pieces used per patient.

**Table 1:** Patient demographic characteristics

Characteristic	Value
Total patients	34 patients
Patient age	38-73 years (median = 53 years)
Figo stage	
Stage 2B	16 patients
Stage 3B	6 patients
Stage 3C	12 patients
Dose to point A	600 cGy
Type of brachytherapy applicator used	Tandem ovoid applicator
Number of gauze roll packed	2-–5 pieces (median = 3 pieces)

The dosimetric comparison results between the VGP plan and the SP are shown in Table 2.

**Table 2:** Comparison of rectum and bladder parameters between SP and VGP plan SP: Standard plan; VGP: Vaginal gauze packing

Metrics	SP Mean ± Std	VGP Plan Mean ± Std	Test p-value
Rectum parameters			
Rectum 0.1 cc (cGy)	607.368 ± 114.836	462.868 ± 114.595	0.001
Rectum 1 cc (cGy)	501.64 ± 88.44	386.57 ± 92.03	<0.001
Rectum 2 cc (cGy)	456.31 ± 79.68	355.02 ± 84.19	<0.001
Rectum 5 cc (cGy)	386.51 ± 68.82	308.06 ± 74.42	<0.001
Rectum 10 cc (cGy)	327.587 ± 59.890	267.221 ± 66.503	<0.001
Bladder parameters			
Bladder 0.1 cc (cGy)	881.59 ± 274.8	877.27 ± 266.72	0.986
Bladder 1 cc (cGy)	702.58 ± 150.19	704.57 ± 181.04	0.865
Bladder 2 cc (cGy)	643.63 ± 132.5	642.98 ± 154.82	0.748
Bladder 5 cc (cGy)	561.57 ± 116.34	552.5 ± 123.41	0.710
Bladder 10 cc (cGy)	487.84 ± 101.76	476.46 ± 102.97	0.648
Volumes			
Rectum volume (cc)	85.93 ± 27.23	80.34 ± 27.52	0.154

The rectum dose of 0.1 cc averaged 607 cGy in the SP group and was reduced to 462 cGy in the VGP group (p=0.001). Similarly, the rectum dose of 1 cc decreased from 501 cGy to 386 cGy, the rectum dose of 2 cc decreased from 456 cGy to 355 cGy, the rectum dose of 5 cc decreased from 386 cGy to 308 cGy, and the rectum dose of 10 cc decreased from 327 cGy to 267 cGy, all showing statistically significant reductions in favor of VGP (p<0.001).

No statistically significant results were found for any parameters of bladder doses (p>0.05). In the comparison of SP and VGP, the bladder doses were 881vs. 877 cGy for 0.1 cc, 702 vs. 704 cGy for 1 cc, 643 vs. 642 cGy for 2 cc, 561 vs. 552 cGy for 5 cc, and 487 vs. 476 cGy for 10 cc. The average rectum volume was 85 cc in the SP group and 80 cc in the VGP group, with no statistically significant difference between them (p>0.05).

Statistically significant reductions in rectal dose parameters were observed: 23.79% for D0.1 cc, 22.94% for D1 cc, 22.20% for D2 cc, 20.29% for D5 cc, and 18.43% for D10 cc.

In all patients, the bladder was filled with the same amount of fluid across all plans to achieve a consistent bladder volume. For bladder dose parameters, no statistically significant differences were observed between the two plans.

## Discussion

This study investigated the impact of VGP on radiation doses to the rectum and bladder in cervical cancer brachytherapy. The results showed that the packing method significantly reduced rectal doses: 23.79% for D0.1 cc, 22.94% for D1 cc, 22.20% for D2 cc, 20.29% for D5 cc, and 18.43% for D10 cc. These findings suggest that VGP is an effective method of rectal protection.

VGP offers a more cost-effective and feasible solution compared to other protective methods like hydrogel injections, balloon applications, and rectal retractors (RRs). For example, in the study conducted by Narukawa et al., the placement of a 7.5 mm-thick hydrogel in patients with prostate cancer resulted in a 10 Gy reduction in rectal doses [10]. Although this method provides greater dose reduction than gauze packing, its cost and availability are limited, especially in resource-constrained settings.

In the study by Khalil et al., the use of spacer injection in proton-based prostate radiotherapy resulted in a reduction of the rectal volumes receiving high and medium doses (73-50 Gy), and a significant decrease in grade 2 and 3 rectal toxicities was achieved [11]. These results suggest that advanced technologies like hydrogel may be more effective for rectal protection in cervical cancer brachytherapy.

Indeed, in a study involving 36 patients, 28 of whom had cervical cancer, it was demonstrated that hyaluronate gel injection significantly reduced the rectum D2.0 cc and D0.1 cc doses [12].

In another study where hyaluronate gel was injected as a spacer into the rectovaginal fossa and vesicouterine fossa during brachytherapy for cervical cancer patients, though a better CTVHRD90 (equivalent dose in 2-Gy fractions (EQD2)) was achieved, no benefit was observed in the bladder and rectum D2.0 cc doses [13].

In a report involving a single patient where hydrogel was injected into the mesosigmoid to protect the sigmoid colon, another at-risk organ in cervical cancer brachytherapy, a significant reduction was achieved [14].

There are also many studies on rectal balloons used as another spacer tool. A recent study reported that when rectal balloon spacers were used in radiotherapy for localized prostate cancer, 90.6% of patients experienced clinical benefit, with an average 25% reduction in rectal V70 doses, and 11% of patients achieved a 100% reduction [15]. However, these balloons require specialized equipment and are more costly.

The use of RRs in prostate cancer reduces intra- and inter-fractional prostate movement and also lowers rectal doses, although their use may be limited by patient comfort and the invasiveness of the procedure [16,17].

In a study by Sud et al., speculum-based vaginal packing (SBVP) was compared to conventional VGP in HDR intracavitary tandem and ovoid brachytherapy for cervical cancer. SBVP reduced bladder D0.1 cc doses by 9.3% without significantly affecting rectal doses, demonstrating its effectiveness for bladder protection and cost-effectiveness [18].

In a prospective study comparing three rectal-dose-reduction methods in cervical cancer, an RR blade, VGP, and a Foley balloon (FB) were used. The results showed that the RR method provided the best rectal dose reduction, while no significant difference was found between FB and VGP [19].

Raiet al. demonstrated that bladder-rectum spacer balloons with VGP (BRSB) reduced D2 cc rectal doses by 12% (7.1 Gy vs. 8.1 Gy) compared to only VGP, although there were no significant differences in D0.1 cc and D1 cc doses [20]. That study suggests that BRSB may be more effective than VGP in reducing certain rectal dose parameters. Also, the finding suggests that simple and cost-effective methods like gauze packing may need to be combined with more advanced devices for optimal results.

Eng et al. reported that the use of an intravaginal balloon in addition to VGP resulted in an average dose reduction of 7.2% (D0.1 cc) to the bladder and 9.3% (D0.1 cc) to the rectum. However, this benefit varied depending on the patient’s anatomy and the placement of the balloons [21].

In another prospective study evaluating the combined use of a vaginal balloon and VGP, the combined approach significantly reduced rectal doses compared to VGP alone but resulted in an increase in bladder doses [22]. In combined use, bladder doses appear to be an important criterion in patient selection.

Xu-Welliver and Lin found that using vaginal-balloon-based packing (VBP) along with patient-controlled analgesia (PCA) during HDR brachytherapy significantly reduced radiation doses to the bladder and rectum compared to VGP. VBP reduced bladder D2 cc doses to 85.7% (compared to 104.8% with VGP) and rectal International Commission on Radiation Units and Measurements (ICRU) point doses to 55.4% (compared to 65.2% with VGP), demonstrating substantial dose reductions [23]. These findings indicate that VBP and PCA improve both patient comfort and dosimetry.

Sawada et al. compared VGP to RR and found that the VGP method reduced D2 cc rectal doses by 8.5% (4.234 Gy vs. 4.627 Gy, p=0.008) and D2 cc bladder doses by 10.9% (5.959 Gy vs. 6.690 Gy, p< 0.001) compared to RR, demonstrating VGP’s effectiveness in protecting the rectum and bladder [24].

In a study comparing the combined use of RR and VGP versus using only RR in tandem and ring applications, the combined approach resulted in lower doses to both the bladder and rectum. Specifically, the ICRU rectum point showed a 13% lower dose, and the ICRU bladder point showed a 10.1% lower dose [25]. These results highlight the dosimetric advantage of VGP, which is easy to apply and cost-effective, even when using more advanced devices.

In a prospective study of 20 patients by Romano et al., VGP was compared to a hydrogen-based packing system (BrachyGel). The study found no clinical dosimetric difference in the bladder or rectum between the two methods. However, in terms of patient and physician comfort, the authors reported that BrachyGel was easier to apply. However, the increase in bladder doses underscores the need for careful evaluation of hydrogel-based systems [26]. In countries with limited economic resources, VGP continues to stand out as the most accessible option.

Despite many methods being studied and newer applications being introduced, an optimal approach for cervical cancer brachytherapy has yet to be determined.

One of the strong features of this study is that it is the first study conducted using external fixators to treat patients without VGP or any other packing method. Another strong aspect is the comparison of plans where VGP was performed and not performed in the same patients. Statistically, dependent groups were compared. However, the weakness is that the applications could not be performed simultaneously, with at least two days between each application. Despite this, it is noteworthy that the rectum volumes were similar. One of the weaknesses is that, due to the absence of an MRI-compatible applicator, the high-risk clinical target volume (CTV) could not be contoured, and doses were defined at the A point in the CT planning.

The bladder volume and the isodoses passing through the right and left A points were also the same, which provided a suitable condition for comparing the effectiveness of VGP. The conclusion that can be drawn from this study is that though new tools are being used for fixation, it is still not possible to abandon packing. This is the first study to highlight this issue. Proper and carefully placed packing significantly alters rectum doses in patients and thus the potential toxicity that may develop. It did not show an effect in reducing bladder doses, indicating a need for further studies in this area. Similar studies with other packing tools would also contribute to this field.

Future research should involve larger patient groups and long-term follow-ups to evaluate packing methods. Combining simple, cost-effective methods like VGP with advanced technologies such as hydrogel or balloon applications may optimize protection for both the rectum and bladder. These combination strategies could be particularly effective in reducing bladder doses and side effects during treatment.

## Conclusions

This study is the first brachytherapy study to include a group without packing. The findings are as follows: VGP is a cost-effective and feasible method that significantly reduces rectal doses during cervical cancer brachytherapy. Even if the stabilizing feature of the fixator is not utilized, VGP is necessary for rectal protection. Therefore, its use should not be abandoned even when an external fixator is used. However, no protective effect on the bladder has been observed, and additional strategies are needed for this purpose. This study contributes to clinical practice by emphasizing safer and more effective brachytherapy applications.
